# Uncovering the determinants of health in deprived urban neighborhoods in Accra, Ghana: a qualitative and participatory reconnaissance study

**DOI:** 10.3389/fpubh.2024.1457682

**Published:** 2024-10-10

**Authors:** Nina Amedzro, Dominic Anaseba, Akosua Gyasi Darkwa, Afua Twumasi, Andrews Ayim, Adelaide Maria Ansah-Ofei, Delanyo Dovlo, John K. Awoonor-Williams, Erasmus Emmanuel Akurugu Agongo, Irene Akua Agyepong, Helen Elsey

**Affiliations:** ^1^Department of Health Sciences, University of York, Hull York Medical School, York, United Kingdom; ^2^Faculty of Public Health, Ghana College of Physicians and Surgeons, Accra, Ghana; ^3^School of Nursing and Midwifery, University of Ghana, Accra, Ghana

**Keywords:** community-based health planning and services, determinants, primary care, public health, social medicine, urban health, Ghana

## Abstract

**Background:**

Delivering primary care services within the context of rapid urbanization and a changing disease burden is a major challenge in sub-Saharan Africa. Rural models of primary care, including the “Community-based Health Planning and Services” (CHPS) programme in Ghana, have shown improved health outcomes. However, adapting these to the urban context has proved problematic. Differences in the determinants of health found in these settings may help to explain the challenges of delivering CHPS in poor urban neighborhoods in Accra. To inform the redesign of CHPS for the urban context, we aimed to understand the determinants driving health and engagement with health services in three informal settlements in Accra.

**Methods:**

This study formed a reconnaissance phase for a subsequent participatory action research study. We used qualitative and participatory methods to explore the influence of wider and proximal determinants on health and the use and perceptions of CHPS. Three transect walks with community leaders across the study settings informed interview guides and the recruitment of suitable participants for key informant and focus group interviews. Using a Framework Approach, we analysed transcripts and reports from these activities and developed themes and sub-themes in participants’ experiences accessing healthcare.

**Results:**

Our findings highlight the importance of wider and proximal determinants of health including physical environment, gender and other social stratifiers including age, ethnicity, religion and disability, on health, health seeking behavior and personal behaviors such as substance misuse, tobacco use and alcohol. Utilization of CHPS was low and seen primarily as a service for maternal and child health. Private providers, ranging from informal drug stores to private clinics, were used most commonly. Community leaders and groups were active, but engagement was limited by opportunity costs for members.

**Conclusion:**

Traditional service delivery packages need to be adapted to include non-communicable diseases driven by risk behaviors such as tobacco, unhealthy diet, alcohol and substance abuse. Assets such as volunteerism and nurses embedded within communities are challenging to attain in complex urban settings, yet other assets exist including occupational associations and a range of informal and private providers that could support delivery of preventive and promotive health care with equitable reach.

## Introduction

Rapid urbanisation in sub-Sharan Africa is changing patterns of health and disease, with rising prevalence of non-communicable diseases (NCDs) alongside persistent risk of communicable disease (1–4). Ghana, with a predicted 60.5% urban population by 2030 (5) is one the most urbanized countries in Africa and typifies these health challenges (6) Within cities there are considerable inequities between households and neighborhoods and aggregate, city-level data masks these differences (7). Analysis of Demographic and Health Survey data from 45 low and middle-income countries found key indicators such as infant mortality and child stunting are significantly associated with living in an informal settlement compared with a formal settlement area, but that the risk of infant mortality was reduced where women had received antenatal care (8). Universal access to essential community-based care is clearly vital within urban contexts in LMICs. With a growing urban population, changing disease burden and limited existing government primary care provision, cities in Ghana are struggling to achieve universal health coverage, which is described by Ghana Health Services as: “timely access to high quality health services irrespective of ability to pay at the point of use” (9).

Ghana’s primary care system is built on the Community-based Health Planning and Services (CHPS) model. The model emerged from progressive learning within the national health system over several decades following the adoption of the Bamako Initiative in 1987 and a series of studies known as the Navrongo experiment (10). Results demonstrated that the strategies were both feasible and improved the impact of primary health care, particularly on child mortality and fertility indicators (11–13). Scale-up of the model has been part of national policy ever since, and the model includes community-based health nurses, volunteers and community engagement to deliver universal access to basic curative care, health promotion and prevention (14). However, despite this success, adapting the model to the urban context has presented many challenges often due to different social and economic structures and the changing disease burden in urban areas. Key challenges include establishing and maintaining support from volunteers; limited low-cost sites for the location of CHPS facilities; staff recruitment and retention particularly ensuring staff are resident in the localities they serve (a principle of CHPS); conducting household visits and outreach when urban residents are at workplaces and, predominantly female, staff fear their safety (10, 15, 16) A review of CHPS by Ghana Health Services found that in the Greater Accra region only 672 of the 834 zones were deemed “functional,” and only 539 of them had basic equipment to provide services (17). These challenges in provision of primary care mean that urban residents, particularly the poorest, rely on a plethora of small private providers and pharmacies where quality and affordability cannot be assured (18).

A number of studies have helped to understand health seeking behavior among those living in informal settlements (6, 8, 19, 20). A recent scoping review characterized the demand and supply side factors driving health seeking behavior in urban informal settlements finding significant challenges in accessing, using and providing health services in these areas (20). The review highlighted the complex interplay of the physical environment of informal settlements with the socio-economic, legal and political, health system, and personal behavioral and experiential factors in influencing perceptions of health needs and the desire for, access to and utilization of health care. Urban poor communities are extremely heterogeneous and the intersections between gender and a wide range of social and economic characteristics influence health and access to health care. This paper presents a qualitative exploration of the health issues and health seeking behavior of urban poor communities in Greater Accra and forms part of a larger participatory action research (PAR) (21) study to develop a CHPS model that can feasibly be delivered in urban areas and meet the needs of low-income urban residents particularly those with vulnerabilities to ill-health, including people with disabilities, substance abuse issues and commercial sex workers.

## Materials and methods

This paper presents our findings from the ‘reconnaissance’ phase of our study. This initial phase is designed to inform a participatory action research (PAR) study by ensuring that the principles and plans for action are grounded in the realities of those affected with the problems under study (22). We used qualitative and participatory methods to explore social structures of poor urban communities, vulnerable individuals and households, their main health issues and identify current health-seeking behavior in order to inform adaptations to the urban Community-based Health Planning and Services (CHPS) model.

### Study settings

The study was conducted in three urban poor communities within Accra, the capital of Ghana, which is located in the south of the country on the coast. These study sites were purposely selected in collaboration with the Greater Accra Regional Health Management team using routine CHPS data and the local knowledge of the neighborhoods, to include: (i) an informal settlement of predominantly first-generation migrants; (ii) a mixed poor/wealthier neighborhood; (iii) a long-established neighborhood of several generations, (iv) different levels of CHPS functionality and (v) different levels of access to alternative health service providers, including referral facilities.

**Figure 1:**
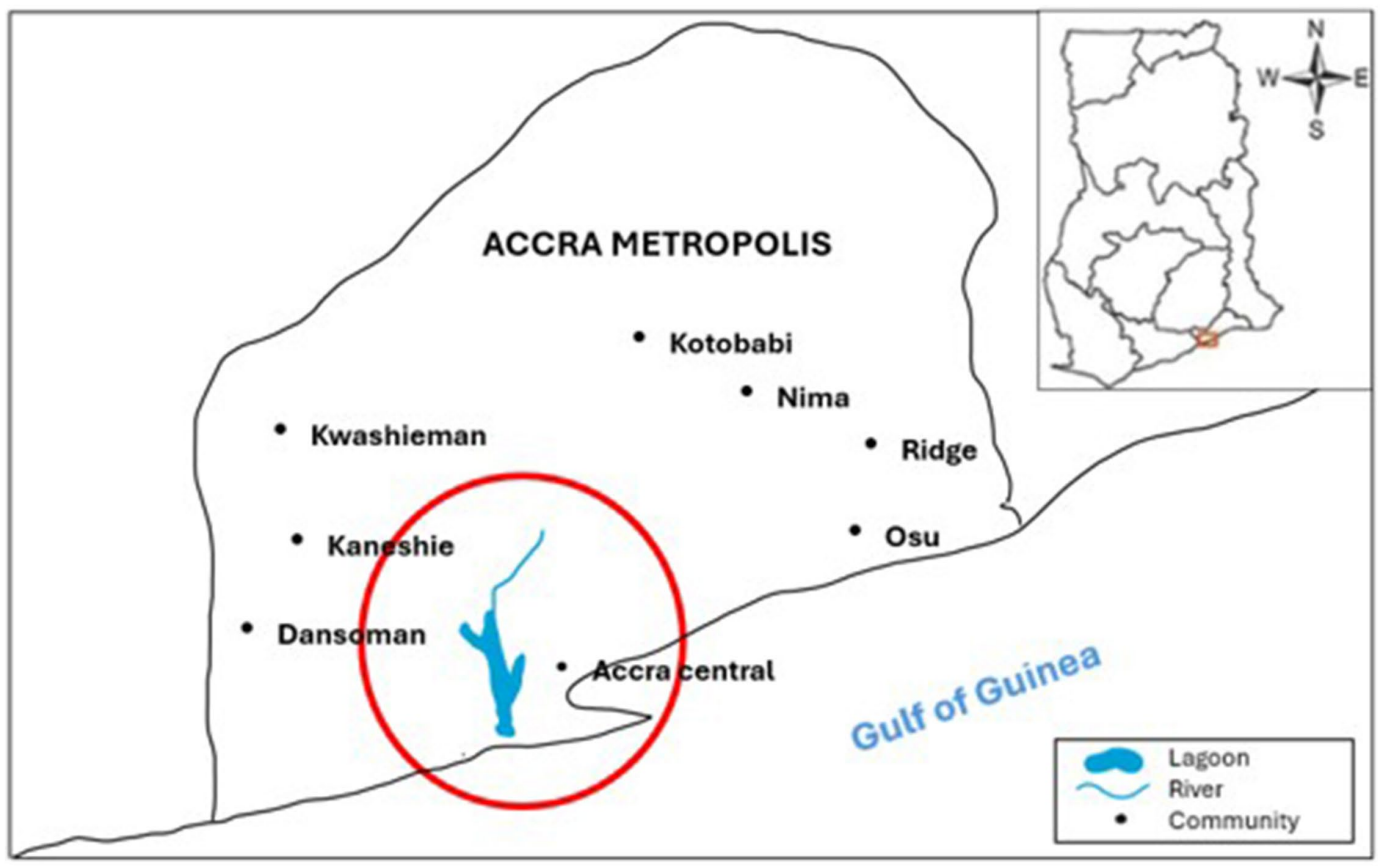
Map of Accra Metropolis showing study settings located within boundary of the circle.

Setting 1 and setting 2 are located within Ashiedu Keteke sub-district, while the initial third setting, known as setting A, is within Adabraka sub-district. Following further assessment, it was decided to replace setting A with an alternative, setting 3, which serves as the headquarters for all six CHPS zones within the Adabraka sub-municipal and is led by the “officer-in-charge” with responsibility for major operational decisions across the zones. Figure 1 shows the general location of the settings within Accra. Table 1 shows information on all of the final three chosen setting characteristics including population, size, main occupations and environment.

**Table 1:** Setting characteristics.

Setting 1	Setting 2	Setting 3
Population: 80–90,000 residents Area: 31.3 hectares Occupations: major markets selling onions, yams, and scrap materials; young females work as Kakayes (head porters); young males work as Okada riders (motorcycle taxi) Environment: long standing community (established 1981) bordering the heavily polluted Odaw River and Korle Lagoon; informal settlement with no drainage systems, persistent flooding during rainy season. Most amenities (water, bathroom facilities etc) privately owned. Pollution from local industry. Population characteristics: Largely youthful, mobile, migrant population from Ghana’s Northern territories, Muslim majority	Population: 18–20,000 residents Area: 37.2 hectares Occupations: fishing; wood/metal work (trunk and oven production); trading in general goods Environment: older formal settlements interspersed with informal. Poorly maintained infrastructure where any exists; amenities privately owned, with water transported in by tanker and sold to residents; local assembly supporting construction of biogas toilets. Pollution from local industry. Population characteristics: majority from indigenous Ga ethnic group, other ethnic groups include Fantes, Ewes, Christian majority, few other religions present	Population: 20,000 to 30,000 residents Area: 34.3 hectares Occupations: market trading of plastic goods and food stuffs; fishing and fish processing Environment: older formal settlements interspersed with informal. Partly bordered by Odaw river; drainage systems inadequate and easily overwhelmed during rainy season; poorly maintained infrastructure; amenities privately owned, but water provided free of charge for the most vulnerable residents via water tank installed by Municipal Assembly. Population characteristics: mainly from indigenous Ga ethnic group, other ethnic groups include Hausas, and migrants mainly from the North. Christian majority;

### Study design

Primary data were collected using a sequential participatory and qualitative approach. Initially, transect walks (23) in the three study sites were conducted, each taking around 2–3 h to complete. These were followed by focus group discussions (FGD) with young people and low-paid workers and key informant interviews (KII) with CHPS staff and community leaders. The FGDs lasted between one and 2 h each, while an interview typically took up to an hour to complete. All data were analyzed to understand the socio-economic and wider context of the slums as well as the health seeking behavior and needs of different groups, particularly those identified by the communities as vulnerable to ill-health.

### Participants

In addition to collecting data to inform the PAR phase, we aimed to build rapport with community leaders and gain a detailed understanding of the context of each setting. To facilitate this, we first identified gatekeepers in each of the three settings. These included official community representatives on local unit committees (the lowest level of local government structure) and the district assembly, older adults with leadership responsibilities from different ethnic groups who are long-term residents and community health workers who are part of the CHPS programme. During the transect walks, other community leaders were identified and included in discussions, particularly religious leaders, youth group representatives and leaders of different occupational groups such as market traders, metal workers and plastic sellers. The discussions during the transect walks highlighted the assets and challenges in each setting as well as any particularly vulnerable groups. This information guided the development of subsequent data collection tools and purposive sampling for the focus group discussions, key informant interviews and individual interviews.

Based on the information obtained through the transect walks we used purposive sampling to select participants for key informant interviews, focus group discussions and individual interviews This allowed selection of participants who were more likely to provide appropriate and useful information (24). Community gatekeepers identified during the transect walk assisted in recruiting participants for interviews. In all, we selected 9 participants for the key informant interviews, made up of 4 health staff and 5 community leaders. Health staff were selected based on their breadth of roles in the provision of CHPS services and their experiences and understanding of the health needs and health seeking behavior of community people. Thus, two front-line community health care providers (Community Health Officer, CHO) and supervisors (CHPS coordinators) at the sub-district and regional level were selected. The community leaders were selected to represent different levels of local government, traditional and religious authorities. They included two Assembly members, one unit committee member, an ethnic chief from the Dagomba ethnic group and a Christian pastor.

Vulnerable groups identified during the transect walks and CHPS zones included people with disabilities, commercial sex workers and substance users. Within all sites, community members highlighted how vulnerability can be particularly acute at certain points in the life course, including for pregnant women, young people particularly those working in the informal sector, and children aged under 5 years and the older adult. To explore the health needs and health seeking behavior of these vulnerable groups we conducted individual interviews with 10 participants, including: two commercial sex workers; two persons with substance misuse disorder; two pregnant women; two mothers with children under the age of 5 years; a disabled mother; and an older person aged over 65 years. One focus group was held with young people and one with female head-porters, known as Kayaye. The individual interviews enabled detailed personal discussions with vulnerable individuals whereas the focus groups were appropriate for groups of peers who were comfortable sharing their views and experiences in a group setting (25).

### Data collection

Data collection took place between May 2021 and October 2022. Reports were written following the transect walks and CHPS zone visits with detailed reflections from the research team. Interviews and focus groups were audio-recorded, transcribed from the Twi dialect and Ghanaian Pidgin English in which they were conducted, and translated into English to facilitate analysis.

### Analysis

We used a Framework Approach (26) to analyze transcripts from the focus groups and interviews, the three reports from the transect walks in each site and CHPS zones reports. In order to aid our identification of a wide range of possible environmental, socio-economic, legal, policy and political, health system and personal behavioral and experiential factors we drew on the framework proposed by Park et al. (20) which includes these multiple domains and their interactions to explain health care utilization. Within each of these domains we used an inductive approach to generate codes and themes to explain our participants’ experiences and perceptions. We used Nvivo 2020 software to organize our analysis; this included setting attributes for each participant based on their age, sex and occupation. Independent coding was done by three of the qualitative researchers on the team, codes were compared, differences resolved and then merged. Framework matrices were produced and exported into Excel to facilitate analysis. In this way we were able to compare themes and sub-themes across sites and by demographic and socio-economic characteristics.

### Data quality assurance and control

To ensure the high quality of the data, the team of researchers from York and Ghana worked closely in developing data collection guides and standard operating procedures for field work. The research team met regularly using virtual platforms to review and revise the guides and ensure interview/discussion questions explored emerging themes and were aligned to the study objectives. Following each round of data collection, recordings were transcribed and translated, and transcripts shared with the research team for review and feedback. This ensured emerging issues and gaps were identified and further explored in subsequent interviews. Following the preliminary analysis of the data, findings were shared with community gatekeepers and members through community workshops. This process of member checking (27) was aimed at validating the findings and triggering discussions within communities to guide the first cycles of participatory action research in the communities.

### Ethical considerations

We obtained ethical approval for the study from the Ghana Health Service Ethical Review Committee (GHS –ERC003/10/20) and the University of York’s Health Sciences Research Governance Committee (HSRGC/2020/409/E). We obtained administrative approval to conduct the study from the Greater Accra Regional Health Directorate, the Sub-Metropolitan and Sub-Municipal Health Directorates of the study areas and the Policy, Planning, Monitoring and Evaluation Division of the Ghana Health Service. Written informed consent was sought from all study participants in key informant interviews, individual interviews, focus group discussions and transect walks. Anonymity and confidentiality of study participants was observed and maintained throughout. The study was funded by the Medical Research Council, United Kingdom.

## Results

Details of the characteristics of participants involved in each of the data collection activities are given in Table 2. Following the overarching domains of physical environmental, socio-economic, health system, political and local government, personal and biological and experiential factors in influencing health needs and health seeking behavior we present the facilitators and barriers to ill-health for different individuals in the three different settings.

**Table 2:** Participant characteristics.

ID	Method	Location	Sex and age group	Role and/or vulnerability as identified by communities
KII0101	Key informant interview	Setting 1	Female 30–39 years	CHO - health service provider
KII0203	Key informant interview	Setting 3	Female 40–49 years	CHPS coordinator- health service supervisor female
KII0303	Key informant interview	Setting 3	Female 50–59 years	CHPS coordinator - health service supervisor
KII0402	Key informant interview	Setting 2	Male 60–69 years	Opinion leader
KII0502	Key informant interview	Setting 2	Male 30–39 years	Community leader/local authority representative
KII0602	Key informant interview	Setting 2	Male 40–49 years	Assemblyman
KII0703	Key informant interview	Setting 3	Male 40–49 years	Assemblyman
KII0802	Key informant interview	Setting 2	Female 30–39 years	Health Service provider
KII0901	Key informant interview	Setting 1	Male 40–49 years	Opinion leader
FGD0102	Focus group discussion	Setting 2	11 Male and Female	Youth group/advocacy for community development
FGD0201	Focus group discussion	Setting 1	10 female	Vulnerable group
TC0102	Transect walk	Setting 2	Male and Female	Community gate keepers
TC0201	Transect walk	Setting 1	Male and Female	Community gate keepers
TC0303	Transect walk	Setting 3	Male and Female	Community gate keepers
II0101	Individual interview	Setting 1	Female 30–39 years	Disabled mother
II0201	Individual interview	Setting 1	Female 20–29 years	Pregnant woman
II0301	Individual interview	Setting 1	Female 30–39 years	Single mother (twins)
II0402	Individual interview	Setting 2	Female 10–19 years	Pregnant teenager
II0502	Individual interview	Setting 2	Female 10–19 years	Teenage mother
II0602	Individual interview	Setting 2	Female 60–69 years	Older adult person
II0704	Individual interview	Setting 3	Male 30–39 years	Substance user
II0804	Individual interview	Setting 3	Male 40–49 years	Substance user
II0904	Individual interview	Setting 3	Female 50–59 years	Commercial sex worker
II1003	Individual interview	Setting 3	Female 40–49 years	Commercial sex worker

## Physical environment

All three study settings had significant environmental challenges to good health with inadequate water, drainage and sanitation facilities, poor waste disposal and inadequate housing.

### Water and sanitation

The extent of informality amongst the housing and living situations influenced the nature of water and sanitation facilities. For example, in setting 1, despite being long established, the settlement is not officially recognized and none of the residents have tenure, or the necessary documentation (leases and building permits) required to access utility service infrastructure. Residents and local landlords are therefore unwilling to invest in improving water and sanitation facilities within their homes and there is no local government provision.

*“We do not have something like a house. Even if somebody has the land to build a house, he is building to rent it out and not to live there by himself. So, it is difficult to see somebody connect water to his room. We only have people who have made public shower and connected the water to for people to use” (KII0901, male, 40–49 years, setting 1).*

Conversely, settings 2 and 3 have grown around established neighborhoods with clusters of informal settlements and slum housing interspersed with more formal housing. While there is a degree of historic infrastructure in these older communities, the conditions and maintenance of services such as water pipes and toilet facilities are still poor. During the transect walks in these communities, leaders explained how many of the households that were once connected to the national grid water supply have since been disconnected or have an infrequent supply.

To address this gap in provision, private providers have stepped in to provide solutions. In setting 2, residents reported their reliance on water transported in by tanker and then sold to residents for drinking, cooking and bathing purposes. Those who are able to afford it are able to purchase bottled or sachet drinking water, known as “Pure Water.” There was also some evidence of local government provision, with a tank provided by the Municipal Assembly in setting 3, with participants explaining that water is then provided free of charge for vulnerable residents. However, for those struggling financially and with no access to any free water sources, residents rely on untreated water from boreholes.

“*…there are some places in the community for over two years now water does not flow through their taps…There is a man at the top there who has dug a borehole, but that one is not safe because it is not treated…Because people are not getting water they are patronising from other sources. You may think it is nothing now but in future you could be creating problems for the community. In this area the tap flows only on Mondays and Fridays” (KII0602, male, 40–49 years, setting 2).*

With no toilet facilities within homes, residents rely on public facilities and again, these are provided mainly by private providers who charge a fee for their use. Again, there was evidence of the role of local government in improving sanitation, for example in setting 2 the local assembly was supporting households to construct biogas toilets. Despite these attempts by local government and private providers, the transect walks and interviews consistently highlighted the inadequacy of such attempts at meeting sanitation needs of all three settings ultimately leaving residents to rely on open defecation.

*“Yes, a lot of people go to the “big bollar” [refuse dump] at the lagoon to do toilet. For them they do not even want to go inside the public toilet…So in the evening like this when you go there you will see some people “freeing” themselves there… and if you get there in the morning you will see faeces everywhere….” (KII0901 Male, 40–49 years, setting 1).*

### Waste disposal

The safe disposal of waste for residents is a growing problem and a network of government agencies, private waste management companies and more informal arrangements exists, working together to remove waste from the city. While waste management companies do not appear to operate in setting 1, they are available elsewhere, and for a flat monthly fee will distribute dustbins to households and empty them on a weekly basis. The “Aboboya system” (a private waste collection service where individuals driving tricycles charge a fee to collect household waste for disposal) is found across the three settings and was preferable due to a “pay as you generate” fee system.

During the rains, poorer households dispose of household waste in the gutters relying on the rainwater to wash it away. Drainage systems are poor where they exist and reliant on open gutters, while setting 1 has no drainage system at all, so perennial flooding is common and exacerbated by obstructions caused by refuse, leading to associated health risks as a result of waterborne diseases.

*“…a lot of them push their rubbish into the gutters and some give them to the Aboboya people. It’s all about money, if you have the money, you pay and they come and collect it but if you do not have the money you push it into the gutters” (KII0502 male, 30–39 years, setting 2).*

Participants across all three settings reported the health impacts of these poor water and sanitation conditions, with gastro-intestinal diseases commonplace among all ages, but particularly young children. In addition to the inadequate drainage systems, poor waste management and sanitation practices, a large lagoon spans the three study settings providing an ideal habitat for mosquitoes and unsurprisingly, malaria, along with gastro-intestinal disease, was reported as the main health concern. The combination of the poor environment, poor housing and hot environments increased people’s risk of exposure. The focus groups and interviews with some of the most vulnerable residents, such as the Kayaye, highlighted that these environmental factors were driving exposure despite adequate knowledge and even provision of bed nets:

*“The common disease to say is malaria…. some will say my room is very hot so let me just sleep outside. So normally they will sleep outside for like two, three days and mosquitoes are everywhere, they can bite you but then we do not think of them. If you tell them to sleep under a mosquito net, they will tell you that if I do not sleep outside I will not like it” (FGD0202, Kayaye, setting 2).*

### Pollution

During the transect walks participants in settings 1 and 2 complained about the polluted air in their communities, explaining that this emanates from scrap dealers burning tyres and producing harmful fumes or sanding down metals and creating clouds of particulate matter. While the transect walk participants associated these with respiratory illness, during the individual interviews and focus groups only a few participants highlighted headaches and respiratory complaints, however this would appear to be because these were seen as relatively minor ailments, and there was limited discussion of the long-term consequences of exposure to air pollution.

### Housing and homelessness

Housing varied across the three sites with different construction methods, often in response to the environment. For example, in setting 1, dwellings were historically small kiosks constructed from wood on top of the soft, boggy ground. Over the years, communities have reclaimed the land by infilling it with stones and other materials, and as finances allow, kiosks are torn down, and buildings are constructed from cement blocks.

In all three settings, rooms and rent are often shared between multiple occupants. Families may share one room, or where rooms are sufficient, men may stay in separate rooms from women and girls. However, overcrowding is common, with typically 5–10 people sharing a room in setting 3, rising to 10–15 people in setting 1. In extreme situations as many as 40–50 people may share a room, operating a rota system to use the space. These issues were particularly apparent in the interviews and focus groups with more vulnerable participants, who clearly articulated the potential for spread of communicable diseases:

*“If your roommate has cholera and is vomiting anyhow, it’s just a matter of time then you will be infected too” (II0101, disabled woman, 30–39 years old, setting 1).*

Overcrowding provides another reason for residents to sleep outdoors, increasing exposure to mosquitos. However, the risks of remaining within overcrowded homes were particularly apparent for women and girls and several of the key informants highlighted this as a factor driving early sexual activity resulting in teenage pregnancy and sexually transmitted infections.

*“Because of the pressure in the homes, how so many of them sleep in the same room, they easily move into relationships too early, somebody is 13 or 14 years, she is out of school, or they are still in school but goes to sleep in their boyfriend’s house because there is space there. They are easily pushed into relationships that’s why we have teenage pregnancies in our community” (KII0502, male, 40–49 years, setting 2).*

Given the unstable nature of informal employment key informants noted that the rent required was often beyond the reach of the very poor leading to homelessness. In addition, migrant labourers and those arriving from outside the city to conduct business would often sleep in shop fronts or outside in the marketplace, where they are at greater risk of robbery and violence, as well as exposure to mosquitos.

*“They sleep outside in someone’s kiosk or shop, in front of someone’s shop. You will see the person put a mosquito net and he is sleeping in front of a shop. We have a big place here we call it “Ship” when you come you will see a lot of people lying there. At first we were all sleeping there because we wanted air, but now the place has become a bedroom for others and you cannot sack them because someone may come from [outside the city] looking for something to do, and when the person does not find anything he may want to sleep over and may come there and you cannot sack such a person” (KII0602, male, 40–49 years, setting 2).*

For those struggling with substance abuse, homelessness was a common experience and the impacts of this on their ability to look after themselves were apparent during the interviews:

*“I sleep in the open. Where I sleep, sometimes people passing by even think that I have mental issues. Some even cry for us… and …sometimes some people can go up to a year or two before bathing.” (II0703 male, substance user, 30–39 years, setting 3).*

## Legal, policy and political

### Lack of tenure

The lack of ownership of land and housing clearly undermined community members’ ability to improve their homes and environment. Out of the three settings, this was particularly the case in setting 1 where community leaders expressed their fears of eviction and how this undermined people’s willingness to improve their own homes and the wider environment, particularly in relation to water and sanitation. However, given the long-standing existence of the community, and despite these fears residents have been able to improve their homes, with the ability to charge rent as an added motivation:

*“At first, we were doing kiosks because the ground was soft (muddy), there was a lot of water underground. But we have been filling it with stones and other materials and now the ground is ‘hard’. As the years go by, we destroy the kiosks and build with blocks if you have the money. And many people now want to live in block rooms rather than kiosks. So, if you have a kiosk and you want to rent it out, the people now prefer the block rooms than [the] kiosks” (KII0901 male 40–49 years, setting 1).*

### Politics driving action

The importance of local politics in stimulating action for improvement came out clearly in the key informant interviews.

*“The women groups we have, but in Accra here they do not have time to organise themselves. Unless it is election time, and we are coming to vote.” (KII0901, male 40–49 years, setting 1).*

Where such political activity was absent, as reported in setting 2, collective action and community organization was non-existent:

*“For this area if you do not have leaders like MPs, you cannot do anything and you can organise a durbar too, but they will not attend” (KII0502, male, 30–39 years, setting 2).*

In addition to triggering community organization, elections were also identified as a catalyst for action to improve the physical environment, including the provision of water tanks and even direct waste collection from homes:

*“…if you do not have the money, you push it into the gutters because the [waste] tank is only one and it’s far from here, that’s our biggest problem here. That time we were going into the Assembly and Unit Committee elections, we used to go into people’s homes and take their “bollar” [waste]” (KII0502 male, 30–39 years, setting 2).*

Outside of election time, several respondents referred to support from assembly members to provide basic food or clothes during festivals, and also during the COVID pandemic, and the role of proactive assembly members in negotiating a reduced fee for waste collection. However, access to this support was clearly more limited when not associated with political patronage with respondents reporting raising concerns about water, sanitation, education and health services with assembly representatives and MPs to no avail, along with indications of empty promises of politicians. The interviews with substance abusers and commercial sex workers highlighted assembly members rarely work to benefit them:

*“Nobody comes to help us sometimes the assemblyman comes to help the junkies I heard he gets money with our name, but he does not bring it to us he does not consider us at all when a junkie dies, he is quick to arrange for an ambulance to come for him there are people in the community that he supports but not us. This morning one of our colleague prostitutes died but nobody has helped. She died in her house after a childbirth” (I11003, commercial sex worker, female, 40–49 years).*

In addition, some Community-based Health Planning and Services (CHPS) staff felt political leaders, including assembly members only take action if payment is offered, or if they happen to have links with the assembly members’ political parties. *“As for the political groups, they are there. They will get some little money, and they do not do their things just for free or do something voluntary for you without you giving something. They will not come if you are not willing to pay them” (KII0303, community health worker, female, 50–59 years, setting 3).*

Nevertheless, assembly members hold authority over local areas and providers must still seek their approval before setting up services. A substance user reflected that a health agency was “sacked” and forcibly removed, despite the perceived benefits they may offer to service users.

*“…we need health screening. The Assembly man sacked a woman who came to provide health screening services just because she did not ask for permission from him. So, the woman took it to the new market area. Even that place it was difficult for some of us to go there. The person who does not use or not addicted may think we are crazy for not seeking health care when we are sick…” (II0703 substance user, male, 30–39 years, setting 3).*

### Government policy and implementation

While there was some indication of government support for infrastructure projects, particularly via the local assemblies, such as water tanks or toilet provision, policies aimed at providing financial protection from health and social costs were reported as facing multiple implementation challenges across all three sites. For example, the national social protection programme “Livelihood Empowerment against Poverty” (LEAP) which offers free enrolment to the national health insurance and aims to provide a quarterly stipend to very poor households, had not been fully rolled out in any of the settings. In setting 3, while community leaders reported sharing a list of vulnerable people considered suitable beneficiaries with LEAP programme organizers, which included the older adult, the chronically ill and the very poor, full implementation across the setting had not occurred. In setting 2, healthcare staff reported having received some basic training on the programme but no further action supporting introduction of LEAP had taken place:

*“We are yet to [receive LEAP]…we went for a training about two months ago… they say they are yet to send that LEAP programme” (KII0802, community health worker, female, 30–39 years, setting 2).*

Similarly, participants from across all categories reported challenges with registering for and then using the national health insurance (NHIS). For some, registration was seen as a laborious process, requiring travel and considerable patience due to long waiting times. The implications are highlighted by one participant, a single mother whose lack of health insurance led her to deliver at home:

*“I went to the hospital, they told me my insurance had expired and when I went to renew it they said the name on the insurance did not match with the name on my Ghana card so I had to go and do it all over again. I kept going back and forth and they were telling me stories about the card being unavailable. It was within those times that I entered into labour and because I did not have the insurance, I did not bother going to the hospital to be charged 2,400 cedis. That is why I stayed and delivered my twins in the house….So, I decided to stay home and pray… this woman who is in my group who knows how to deliver babies she delivered me in the house” (II0301, woman, 20–29 years, setting 1).*

Recent improvements to the NHIS registration system include an option to renew using a smart phone. Several of our younger participants were aware and able to take advantage of this efficiency: “*But there is something which has made it very helpful for the renewal. Previously you had to go there (the health insurance office) but now we use our phones to renew, it is very helpful for us” (FGD0101, youth group, setting 1).*

However, for the very poor, covering the cost of registration for NHIS was not possible as was the case for this disabled mother: *“I have the health insurance, but it has expired, and I do not have the money to renew it” (II0101, female, 30–39 years, setting 1).*

## Socio-economic

### Economic hardship and opportunities

The economic challenges of life in the three settings were evident with high levels of financial insecurity undermining health and ability to seek health care. Work is low paid, highly gendered, insecure and subject to seasonal variation, making income difficult to predict and requiring both men and women to work long hours. Many respondents described their financial vulnerability, with recent wider economic crises undermining their abilities to maintain their livelihoods and look after their dependents:

*“Initially the business was very good but now everything has gone down….We buy everything here, from food to going to toilet and even bathing. Therefore, it is very difficult” (FGD0201, Kayaye, female, setting 1) and “When we do not work too, how can we take care of our sons and children?” (FGD0102, male, youth group, setting 2).*

Community location and proximity to markets and coastal areas influences the type of work available. Setting 1, with its well established and bustling markets attracts kayayes (head porters) who are commonly female migrants from the north of Ghana. Work for young men appeared to be more available in settings 1 and 2 particularly due to the markets, which provide transport opportunities, either of passengers (Okada riding) or goods or waste on tricycles (Aboboya riding). However, attempting to gain more stable work seemed to be a hopeless venture due to the stigma of living within an informal settlement:

*“If you go everywhere in Ghana, from North to South, East and West in Ghana, Odododiodio has been painted black, that one, we are violent; two, we do not respect; three, we are robbers, thieves – that if you employ us into your organisation we will take your things and run away….If they give you a form to fill or you go for interview, they will ask you of where you come from, they will not even ask of your name they ask, where are you coming from? and once you say you are from Budor, they will say I will call you and that is the end, you can wait 30 years they will not call” (FGD0102, male, youth group, setting 2).*

For women of all ages, particularly those widowed or separated or escaping financial hardship in their rural home-district, commercial sex work was a viable survival strategy. The interviews with sex workers highlighted the impact of the work, and its stigma, on women’s mental as well as physical health.

*“Yesterday my daughter asked me: “Mummy, I do not know where you work. You do not take us there, you only give us your friend’s number to call you when we cannot reach you but we need to know where you work.” After she said that I sent her on an errand, and I began to cry because I do not want my children to ever find out that I am into prostitution.” (II0903, woman, commercial sex worker, 50–59 years, setting 3).*

In all three sites, socioeconomic factors played a significant role in influencing health seeking behavior. The absence of adequate childcare in particular was challenging for those without family or other external support and was frequently raised in the interviews with women. This clearly impacted not only on their ability to seek healthcare for their own needs but also for that of their children, due to long working hours which were a barrier to attending clinics or receiving visits from healthcare workers.

*“She cannot even go there, they will not think of going there, they are thinking about the business and how it is moving” (KII0901, male, 40–49 years, setting 1).*

### Diversity and support through kinship

The three study communities are typically dense, with transient populations as people move to other locations for work, housing or due to evictions and new migrants arrive from other regions of Ghana and neighboring countries. Communities are mostly youthful, and while many multi-generational and extended families were evident during the interviews and transect walks, it was also clear that single-parent families and individuals with loose family ties were also common. Despite the challenges of these changing social structures and many different people from different ethnic and religious groups living together, with language barriers and practices, respondents, particularly young people, reported that in general social relations are good.

*“…we have many tribes here, but most of us are Gas… Even not only the tribes, we have foreigners also here. So, we all cooperate together, we do not have any bad intentions for them” (FGD, youth group, setting 2).*

Across the three sites there were multiple mentions of ethnicity playing a critical role in social mobilization and support, with people aligning with those from their own ethnic groups. These bonds were frequently identified as providing crucial support during times of ill health or hardship with those of the same ethnic group, particularly ‘chiefs’ will contribute financially to medicines and other expenses or provide accommodation:

*“Look at my place here, it does not look like a chief’s palace, but I am inside it! Here used to be clean but look at what you are seeing here, they are like scraps. They do not have anywhere to relocate, and they are my people, they have brought their goods here, can I sack them?” (KII0402 male, 60–69 years old setting 2).*

“*When you get here for the first time at least you get somewhere to stay and sleep. Sometimes we do meet our colleagues from the same place or community… When you tell them about your plight, they do very well to help you especially when they realise you speak the same language with them” (FGD0201, Kayaye, female, 20–35 years, setting 1).*

However, there were clear examples of participants reporting ethnic and religious tensions and strong kinship relationships. While this offered some advantage to those offered opportunities as a result of ethnicity, this also led to divisions and feelings of injustice, particularly regarding employment opportunities:

*“Sometimes, they try to be tribalistic: I will like to pick only Asantes to work for me, but in Budor electoral area, we do not have only Asantes, we have Gas who are more than the Asantes here. But they will go somewhere like Agege (another community) and go and bring Asantes to come and work for them. That is not fair….” (FGD0102, male, youth group, setting 2).*

### Community structures, support and social capital

Occupational associations were common, and during the transect walks a range of associations were identified in each setting and included representation for hairdressers, dressmakers, kayayes, fruit and vegetable sellers, yam and onion sellers, trunk manufacturers and traders in scrap metals and plastics. The community health workers highlighted that some of the associations supported their members through cash or in kind during key life events, such as births, deaths and marriages. However, for others, particularly the commercial sex workers, the role of the associations was more for social support and safety:

*“We have “manye,” like a Queen Mother. She’s the leader of this place. There are about 70 of us here and she’s the queen. She does not do much. She’s the replacement for the landlord to settle disputes and any other issues or problems that you have you can take it to her for advice. And we also have a ‘police’, I am the police of the Sisters (Commercial Sex Workers). You know, usually places where you have sisters or prostitutes there’s always fights, violence and other things so we put these things in place to prevent people from fighting.” (II0103, commercial sex worker, female 50–59 years setting 3).*

People with disabilities and the older adult were typically not in employment and tended not to belong to any external associations or groups, therefore increasing their vulnerability due to a lack of advocacy.

Energy for community organizations was clear among the young people interviewed and they reported establishing their own association to support the welfare of participants, reduce elements of unwanted behavior and represent the community in a positive light. However, one Assemblyman reflected on the transient nature of some of these groups, particularly given the limited time and opportunity costs of participating in community organizations:

*“The youth will decide to form a group so that they can help each other. They will create the thing nicely but tomorrow they will not be there” (KII0602, male, 40–49 years, setting 2).*

Communities have diverse and complex leadership structures revolving around groups and individuals with specific interests in the groups they lead and the development of the community as a whole. Numerous leaders may represent political or religious groups, economic activities (trade/craft-associations), youth or organized social groups, as well as family heads.

*“We have leaders everywhere… In our community here, we have about sixteen tribes and all the tribes have chiefs who are their leaders” (KII0901, male, 40–49 years, setting 1).*

Leaders mobilize social support systems, resolve conflict and unwanted behavior within their group, and advocate for support and development from appropriate partners. They able to organize their members and raise funds to support with healthcare costs when an urgent need arises, as described by this leader of a trunk manufacturing association:

*“We were cutting metal plates, a small boy was sitting on his bike riding, he fell, and the plate cut his hand, we took him to Korle Bu [public hospital] and they said we should pay about 2000 cedis or so. I called people and they contributed small, small and we took care of the boy. So, if anything happens we contribute as a group and do it” (KII0402, male, 60–69 years, setting 2).*

## Religious and cultural factors

CHPS workers were able to share how religious and cultural beliefs were found to influence many health related decisions. In setting 1, this was particularly challenging in relation to family planning and vaccination, including through the influence of local chiefs:

*“You will go, and you are talking about family planning, and the chief will say I have 4 wives and I want 20 children…I remember I spoke to one chief and he told me to stop what I am saying because if he sees that his wife is doing family planning, what he will do to the wife, ehh? So, what is this leader going to tell others, to come and do family planning? It’s hell no” (KII0101, community health worker, female, 30–39 years, setting 1).*

Religious institutions also acted as an asset within the community, as explained by substance misusers who reported accessing churches and mosques as they provide food and other social support, particularly during religious festivals and holiday seasons.

*“(Rhema Church) bring us food, that’s where I have made up my mind to go. The Apostle comes to talk with us, that’s why we have decided to go there” (II0703, substance user, male, 30–39 years setting 3).*

The skills and knowledge of traditional herbalists in treating illness were widely regarded across the settings, particularly amongst those in less well-paid work with fewer funds to seek healthcare elsewhere. Observations from the transect walk in setting 2 highlighted a number of traditional practitioners and for some conditions, spiritual and traditional herbal medicine was seen as more appropriate than orthodox treatment:

*“It’s not all the sickness that can be detected in the hospitals. The traditional medicine practitioner (herbalist) plays a part in treating people here, they can treat some of the sicknesses. You can always get a herbalist to attend to you when you have any health problem” (FGD0202, Kayaye, female, 25–35 years, setting 1).*

## Cognitive and experiential factors

This theme focused on participants’ experiences of health care, and the overriding perspective in all three settings was that public health care was not accessible, health workers were frequently absent, medicines were not available, and quality was poor.

*“People in the Electoral area refuse to attend the hospital for health services because there is a long standing perception that if one is sick and you visit the hospital for health services for any serious condition, you are likely to die from the condition because of inadequate attention that you are likely to receive from service providers…The private health facilities are perceived to give better care because they want good name for the facility so people patronise their services when the need arises” (KII0703, male, 40–49, setting 3).*

Most people were aware of the local clinics and hospitals available in their area, however, experiences of clinics in setting 2 were particularly poor, with participants, especially younger participants reporting healthcare staff to be disinterested, absent or not available, especially in the night:

*“…when it comes to our health care, it is very bad. There will be a case and the doctor and the nurse in the clinic will not attend to the patient. They will be saying that go and do A, or go and do B, while the person is dying instead of taking care of the patient. Even in the night, you will go to the hospital, and they will say there is no doctor, but the doctor is sleeping in the office” (FGD0102, youth group, setting 2).*

The combination of these perceptions and the limited time available to travel to facilities far from work or home, meant a high use of private pharmacies was identified across participant groups. A plethora of small drug shops were identified during the transcripts, clearly meeting the high demand within the communities. CHPS staff report being aware of unregulated health facilities operating at drug stores, which can have negative health implications for those attending them, and also on the work of CHPS:

*“They have secret ones [health facilities], some of which we are not even aware. They will tell you it’s a drug store, but it is actually a clinic…. They do what clinics do, they even give infusions and all that…. it’s really affecting our work. Sometimes some diseases are above them and instead of taking it to the nurses so we can also refer, they will not. They will treat them and by the time they get to the hospital it’s too late. Maybe if they had come earlier their bill would have been GHC 100 but they will be doing “try and error”* (*KII0101, community health worker, female, 30–39 years, setting 1*).

The perception of the CHPS programme was primarily as a service for mothers and children, providing routine appointments to monitor and weigh children, provide immunizations, and promote health through educational events. Other services provided by CHPS did not seem to be widely acknowledged, and none of the male interviewees reported consulting with the CHPS nurses in times of ill health. The low community awareness of available CHPS services may in part be down to the fragile relationships between healthcare workers and key community members, often exacerbated by the high turn-over of staff and the lack of locally resident CHPS nurses:

*“Well, as the lady left (retired nurse) …you people keep changing the people. The first people that the lady brought, all of them know me…now the people that are coming there do not know me and I do not know them…they do not know who has allowed them to be sitting there… I cannot introduce myself to them so far as they have not come to me” (KII0901, male, 40–49 years, setting 1).*

## Personal and biological factors

### Changing behaviors

There was some indication of changing diets in the urban context. In part, this was seen as another consequence of inadequate housing and overcrowding which led to the lack of space for cooking. Food is often cooked outside in the open, making it a labor intensive and more expensive option, and increases the risk of fire. Time and space are at a premium, so many opt for the convenience of buying food from local street vendors or “chop bars” rather than cooking vegetables and more traditional dishes at home. However, while respondents mentioned high blood pressure and diabetes or ‘sugar level’, none connected these changes in practices to any rise in non-communicable diseases, instead seeing these as an inevitable part of ageing.

A common issue raised by key informants and during the individual interviews was the increasing use of tobacco, alcohol and marujana and other illicit drugs. This was linked to anti-social behavior, something which was particularly highlighted by this respondent with a disability:

*“Yes, people smoke here. ….People get drunk and behave in weird and annoying ways. One of them even hit me this morning. I asked him to apologise, and he started complaining. The best I could do was to prevent things from escalating by keeping quiet*” *(II0101, female, 30–39 years, setting 1).*

The link between the socio-economic realities and behaviors harmful to health were clearly articulated by the substance users interviewed:

*“I was a driver’s mate at first, with the aim of becoming a trotro [mini-bus] driver. But I was not able to acquire a driver’s licence for the job… I now find myself in this lifestyle due to bad friends….I am talking about the drugs lifestyle” (II0803, male, substance user, 40–49 years, setting 3).*

### Increasing vulnerability through old age and disability

Older adult people may have multiple comorbidities and having initially moved to the city to work, have few relatives to provide support. They are at risk of becoming increasingly isolated, reliant on others for money and their basic needs.

*“I go back home to take my bath and stay indoors the rest of the day… I used to sell but because of my sickness I cannot do that again, so I am always at home now… I have left my [food selling] business with someone who sells for me now…. She always tells me the food was not patronised. She brings me small money…. The money…is not enough, but what can I do? People also give me money as a gift, too” (II0602, female, 60–69 years, setting 2).*

People with disabilities struggle to find employment and reported begging for money, receiving negative treatment from members of the community and also from health workers which has a negative effect on mental health:

*“I just want jobs to be made available to us. That way, we are not burdens on others and will not be treated as burdens. I’m also pleading with my fellow disabled persons to get jobs… Stop begging on the streets so we can be accorded some respect…as for we the disabled ones, they do not count us as part of human beings because of the treatment they give us. I mostly cry because of that. I sometimes feel like killing myself because of the treatment I receive” (II0101, female, 30–39 years, setting 1).*

## Discussion

Our findings highlight the fundamental importance of the wider determinants and more proximal determinants of health. These determinants shape impacts on health and access to health care through a prism of gender and other social stratifiers such as age, ethnicity, religion and disability. The framework developed by Park et al. (20) provides a useful entry point to identify and structure understanding of these determinants. Within the context of our findings, the physical environment and socioeconomic factors were particularly important in shaping health and access to health care. Within the physical environment domain, housing, water, sanitation and waste were consistently raised as undermining health and well-being. Understanding these elements would seem to require further delineation than offered in the Park et al. framework, and it is interesting to note that Abascal et al.’s (28) review which looks more toward combining data (surveys and earth observation) to understand deprived urban neighborhoods to inform planning, distinguishes between elements of the physical environment to identify particular challenges with housing, unplanned urbanization, contamination and infrastructure. The socio-economic category is also further broken down into socio-economic status and social hazards and assets as well as city governance (28).

Our findings show that the domains are clearly interlinked with physical environmental challenges such as overcrowding and inadequate housing combined with personal vulnerabilities. This was particularly the case for young women with overcrowding exposing them to sexual abuse and risk teenage pregnancy. Women’s living conditions have been identified as a determinant of women’s mental ill health (29), and while our respondents did not explicitly mention depression or other forms of mental ill-health, most likely due to stigma and limited knowledge of mental ill health in the Ghanaian contexts, the impacts on well-being were clear in our own findings. The socio-economic domain also clearly intersects with the personal and behavioral domains. For example, while all struggle to find a daily wage, this appears particularly acute for people with disabilities who have little option but to beg and older people, whose situation was worsened due to the lack of any extended family to care for them within the slum. What little evidence there is of the determinants of health for the older adult and people with disabilities in slums shows an intriguing picture with occupation not being a statistically significant determinant of health and older people dependent on their own sources of livelihood less likely to report a disability (30). This may reflect out-migration and higher mortality for older people with a disability in slums.

The socio-economic domain was also associated with personal behaviors, these more proximal determinants, such as alcohol, tobacco and substance abuse have been associated with a range of family, peer and commercial determinants in a number of studies in informal settlements. For example, a cross-sectional study of alcohol use in Kenya’s slums identified additional social determinants such as a culture of peer drinking, a history of family drinking and dysfunctional families as increasing the risk of problem drinking (31). Urban social norms, peer networks and greater exposure to tobacco advertising have all been identified as determinants of tobacco use for women migrating to cities in South Africa (32). In Pakistan, a small cross-sectional study in slums found the wide availability of drugs, high tobacco use and child labor associated with high prevalence of substance abuse (33).

Our findings highlight the limited use of primary care, particularly the Community-based Health Planning and Services (CHPS) programme, which was seen as a service primarily for maternal and child health. The role of private providers, from informal drug stores to private clinics came out clearly as the most commonly used source of health care, and this is consistent with many other studies conducted in urban areas in LMICs (18) and in informal settlements in Ghana where cross-sectional studies have found that over 70% of residents had not visited a health facility in the last 5 years, with 60% accessing treatment from drug stores and 33% from hospitals (34). Use of health care in slums is highly inequitable, but even better off households are likely to experience catastrophic health expenditure (35). Our findings indicate that many, particularly older residents without access to smartphones and the poorest, are unable to benefit from Ghana’s NHIS, thus increasing their exposure to financial risk and another barrier to accessing health care.

This study was conducted as a reconnaissance phase to a PAR study in Accra with the aim of improving CHPS provision in deprived urban areas. This focus on understanding determinants to trigger action necessitates careful analysis of the wider determinants, particularly to identify any assets that may mediate the influence of the determinants on health equity. The role of community leaders, groups and associations was a particularly important finding and reflects results of other studies in sub–Saharan Africa which identify the importance of associations in slums, even when levels of crime and socioeconomic disadvantage undermine community organization (36). While members of these groups may struggle to find time or resources to fully support each other, they do provide a valuable asset through which community health initiatives such as CHPS can work. A key feature of CHPS is the use of volunteers, working closely with the community health nurses who are housed within the communities they serve and this, coupled with high levels of community engagement has been associated with the programme’s success in improving maternal and child outcomes (10, 37). The barriers to these two tenets of the CHPS programme are clear in all three settings described in our findings and these have been highlighted in evaluations of CHPS in the urban context (15).

Our findings suggest that careful consideration of the wider determinants of health and access to health care can provide insights into the effective reshaping of the CHPS programme to respond to the health needs, behaviors and structures of informal settlements. Tapping into the potential of linking with private and informal providers could enable closer reach into these communities. Such approaches are being explored in similar contexts in Nigeria (38). Adapting outreach and home-visiting strategies to instead focusing on places of work could overcome the opportunity costs that daily-wage earners face in accessing health care. Reshaping the focus of services delivered to support healthy behaviors and protect vulnerable residents from tobacco, substance abuse, alcohol and unhealthy diets would remain in line with CHPS’s focus on preventative health and help to address the rising tide on non-communicable diseases.

## Strengths and limitations

Several of the communities, particularly the community leaders, had been subject to multiple interactions with researchers and others gathering data in a purely extractive way. This created challenges in initial interactions, and building rapport took time. The use of PAR as a subsequent approach to facilitate change helped in building trust with community leaders. Transect walks were also a valuable method for building rapport whilst also building a strong understanding of the context of each setting, and this approach can be seen as a strength within the study.

The data collection period partially overlapped with COVID-19 restrictions on public meetings, and this limited some of the focus group and wider community meetings planned as part of the study. However, individual interviews were all held face-to-face, and this method proved particularly effective in enabling participants, even those who faced considerable stigma such as substance users and commercial sex workers, to talk openly about their lives and experiences.

## Conclusion

Health and access to health care are strongly determined by the physical environment and socio-economic situation of informal settlement residents and these determinants are shaped by gender, age and disability among others. Improvements to living conditions should include reducing overcrowding and improving access to affordable safe drinking water and must be prioritized to reduce the burden of communicable disease. Innovation could be an important facilitator for change, and steps to address poor sanitation by introducing biogas digester toilets show potential. However, wider rollout is not planned, and barriers include initial outlay and ongoing maintenance costs. Traditional service delivery packages need to be adapted to include non-communicable diseases driven by risk behaviors such as tobacco, unhealthy diet, alcohol and substance abuse. Assets such as volunteerism and nurses embedded within communities are unattainable in these complex urban settings, yet other assets exist including occupational associations and a range of informal and private providers that could support delivery of preventive and promotive health care with equitable reach.

## Data Availability

The original contributions presented in the study are included in the article, further inquiries can be directed to the corresponding author/s.
